# Caulocorea

**Published:** 1885-07

**Authors:** 


CAULOCOREA.—Often we have met with cases termed "female weakness" that have been difficult to relieve, in which there is an atonic condition of the uterus and its appendages, as shown by the prolapsed womb, bearing down pains and pain in the back, leucorrhœa, general debility, etc. In such cases the only thing needed is a tonic to these parts. Caulocorea is a fine stimulant and tonic in cases where a person is easily prostrated.

It is better for palpitation of the heart and nervous headache than any other remedy, and for colic or pain in the bowels, given every few minutes, it is preferable to opium, as it leaves no bad effect. For painful menstruation it is a specific.

Dr. S. M. Smith, of Lima, Ohio, says : "Caulocorea is certainly the *sine qua non* of all remedies."

